# Experimental Analysis of IPMC Optical-Controlled Flexible Driving Performance under PLZT Ceramic Configuration

**DOI:** 10.3390/s24175650

**Published:** 2024-08-30

**Authors:** Yafeng Liu, Pingmei Ming, Jianhui Chen, Chenghu Jing

**Affiliations:** 1 School of Electromechanical Engineering, Henan University of Technology, Zhengzhou 450001, China; chhjing@haut.edu.cn; 2 School of Mechanical and Power Engineering, Henan Polytechnic University, Jiaozuo 454003, China; mingpingmei@163.com; 3 Shitai Industrial Corp Ltd., Taizhou 317100, China; chenjianhui@shitai.com.cn

**Keywords:** IPMC, PLZT ceramic, optical-controlled, flexible driving

## Abstract

Ionic polymer metal composite (IPMC) is regarded as the mainstream application material for achieving flexible driving technology in various engineering fields. In this article, aiming at the non-independence of the current IPMC electric driving method, an IPMC optical-controlled flexible driving method based on the photoinduced effects of lanthanum-modified lead zirconate titanate (PLZT) ceramic is proposed. To this end, a mathematical model for IPMC optical controlled flexible driving is built on the basis of the photovoltaic characteristic of PLZT ceramic, and the driving performance is experimentally analyzed through different lengths of IPMC under the excitation of different direct currents and light intensities. From the analysis and experimental results, when PLZT ceramic is irradiated by different light intensities, the output deformation of IPMC increases with increases in light intensity, and finally reaches a stable state. Moreover, the actuation curves obtained by light excitation and direct current excitation are consistent, and the motion coefficient reflects the driving performance more accurately. In addition, using light energy as an excitation source to drive IPMC not only provides new ideas for its development in the flexible driving field, but also provides a theoretical basis for its practical application.

## 1. Introduction

Ionic polymer metal composite (IPMC), as an electroactivity smart material, has become one of the most promising application materials for flexible driving technology due to the advantages of its light weight, strong flexibility and low driving voltage, etc. [1]. However, IPMC driving usually requires one external power excitation device, resulting in non-independence; this also brings electromagnetic interference and other problems. Lanthanum-modified lead zirconate titanate (PLZT) ceramic has outstanding photovoltaic characteristics [2], being able to convert light energy into electricity and displace the external power supply excitation device to drive the IPMC. Thus, it can not only solve the problem of electromagnetic interference caused by non-independence, but has the advantages of non-contact and remote control. Therefore, the IPMC optical-controlled flexible driving method under a PLZT ceramic configuration has great application potential.

In view of the excellent characteristics of IPMC, scholars have made great progress in the research of its application in the fields of bionics, medical treatment and aerospace. Martinelli et al. [3] outlined the latest trends in the use of different 3D printing technologies to produce electrically responsive IPMC devices for use in various fields from biomedicine to soft robotics. Li et al. [4] designed and prepared a venus flytrap imitation robot driven by IPMC, which can realize repeated opening and closing actions. Ma et al. [5] simulated biological motion based on IPMC and developed a series of bionic robots such as bionic flowers, bionic vines and bionic dragonflies with high-frequency wing flapping. He et al. [6] designed the interventional catheter with square-rod IPMC, realized active guidance, and successfully completed an in vitro simulation experiment of interventional surgery. Lee et al. [7] developed an intelligent and non-invasive IPMC throat sensor, which can recognize coughs, grunts, swallows and nods. Horiuchi et al. [8] developed a dynamic adjustment intraocular lens driven by IPMC that can be inserted into the eye, successfully meeting the demand for visual adjustment. Wang et al. [9] proposed a cantilever beam IPMC soft driver for drug delivery devices that is driven primarily by an external radio-frequency (RF) magnetic field. Feng and Liu [10] proposed an engine that uses four flat-film structure ion-polymer–metal composite (IPMC) actuators to achieve up and down motion.

Over the years, more achievements have been made in the study of the multi-field coupling and photoinduced-effects of PLZT ceramic. Huang et al. [11] introduced the influence factors of temperature changes into the mechanism analysis of multi-field coupling, and proposed and established a new multi-field coupling model for PLZT ceramic. Chen et al. [12] attributed the photorestrictive effects of PLZT ceramic to strong photoinduced electron-lattice coupling and extended it to the visible light range. Sharma [13] constructed a temperature-dependent constitutive equation including piezoelectric coefficient and dielectric constant on the basis of the multi-field coupling of PLZT ceramics, and modified it using a finite element model. Kumar [14] investigated the effects of sintering temperature on the dielectric properties, ferroelectric properties and energy storage properties of PLZT ceramic. An et al. [15] produced a PLZT micro cantilever using a micro and nano-manufacturing process based on the effects of phase transition-induced strain and the sharp phase switch of antiferroelectric materials. Liu et al. [16] proposed an optically controlled variable damping system on the basis of PLZT ceramic and electrorheological fluid. Lu et al. [17,18] developed a lunar dust collector and a crawler automatic dust removal device, respectively, due to the photoinduced properties of PLZT ceramic. Liu et al. [19] proposed an opto-electrostatic hybrid driving method based on PLZT ceramic.

Although based on the photovoltaic characteristics of PLZT ceramic, research on PLZT ceramic as a driving source has been conducted extensively; however, studies of the IPMC optical-controlled flexible driving method under a PLZT ceramic configuration is still deficient, and lacks a corresponding theoretical basis and experimental verification. In this paper, the new mathematical model of IPMC optical-controlled flexible driving is established. Then, the driving performance is tested and analyzed by a series of experiments. For different lengths of IPMC, taking direct current and light intensity as the excitation source, respectively, the curves of the output deformation are obtained and the fast response is acquired.

## 2. Mathematical Analysis of IPMC Optical-Controlled Flexible Driving

### 2.1. Equivalent Electrical Model

When PLZT ceramic is illuminated by a UV light source, a photovoltaic voltage is generated at both ends of the electrode. For the electrical model of the PLZT ceramic, it can be thought of as an equivalent circuit composed of a constant current source, with resistor *R*p and capacitor *C*p in parallel [20], and the photovoltage of this equivalent circuit model is:

(1) $$U_{p}=I_{p}R_{p}(1-e^{-\frac{t}{R_{p}C_{p}}})=U_{s}(1-e^{-\frac{t}{\tau }})$$

where *I*_p_ is the photocurrent, *U*_s_ is the photovoltaic voltage of the PLZT ceramic, τ is the time constant and *t* is the irradiation time.

**Figure 1:**
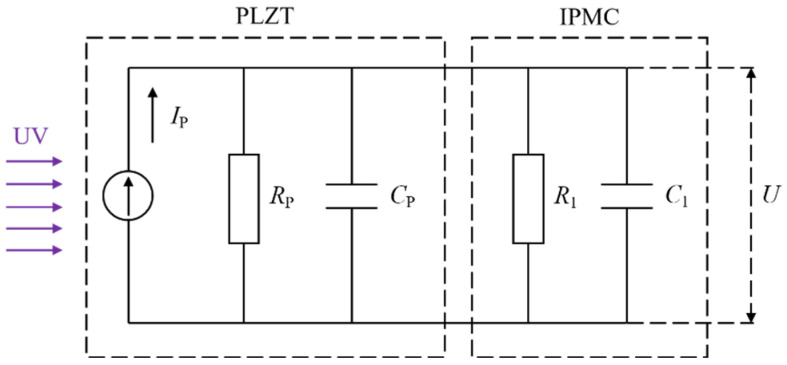
New equivalent electrical model.

For the optical-controlled flexible driving method proposed in this paper, when IPMC is used as a driving load, resistor *R*_1_ and capacitor *C*_1_ can be thought of as being in parallel at both ends of the PLZT ceramic; so, this new equivalent electrical model can be constructed as seen in Figure 1.

From Figure 1, the new capacitance *C* and resistance *R* of the entire circuit can be presented as:

(2) $$\left\{\begin{array}{l} C=C_{p}+C_{1}\\ R=\frac{R_{p}R_{1}}{R_{p}+R_{1}} \end{array}\right.$$

Then, the IPMC optical drive voltage *U* can be expressed as:

(3) $$U=I_{p}\frac{R_{p}R_{1}}{R_{p}+R_{1}}(1-e^{-\frac{t}{\tau^{\prime }}})={U_{s}}^{\prime }(1-e^{-\frac{t}{\tau^{\prime }}})$$

where τ′ is the new time constant, it can be presented as:

(4) $$\tau^{\prime }=\frac{R_{p}R_{1}}{R_{p}+R_{1}}(C_{p}+C_{1})$$

### 2.2. Mathematical Model

**Figure 2:**
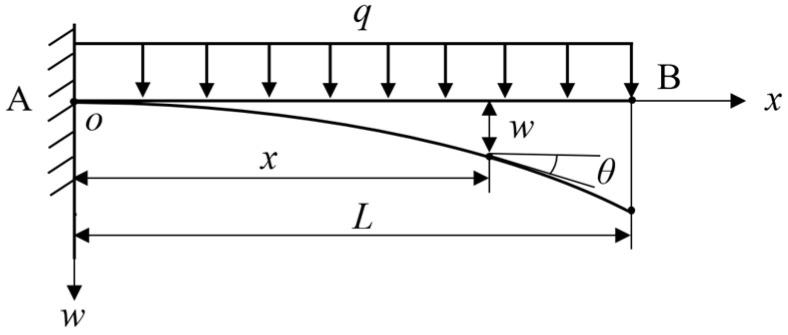
Simplified mechanical model of the IPMC load mechanism.

When PLZT ceramic is illuminated by a high energy UV light source, the photovoltaic voltage is generated to drive the IPMC load mechanism and the driving force is produced in the IPMC as well as a continuous distribution of loads along the thickness of the polymer layer. Therefore, the IPMC load mechanism is simplified and can be regarded as a cantilever beam with one end fixed. The cantilever beam bears a uniform load, and the force direction is perpendicular to the plate direction. *L* is the length of cantilever beam AB, *q* is the distributed force and *θ* is the angle of torsion. The specific mechanical simplification is shown in Figure 2.

To establish the oxw coordinate system, take any position coordinate as (*x*, *w*), and list the bending moment equation as follows:

(5) $$M(x)=-\frac{1}{2}q{\left(L-x\right)}^{2}\;\;\;(0\leq x\leq L)$$

Bring the above equation into the small deflection differential equation and integrate continuously to obtain:

(6) $$EIw^{\prime \prime }=-M=\frac{1}{2}q{\left(L-x\right)}^{2}$$

(7) $$EIw^{\prime }=EI\theta =-\frac{1}{6}q{\left(L-x\right)}^{3}+C$$

(8) $$EIw=\frac{1}{24}q{\left(L-x\right)}^{4}+Cx+D$$

By substituting *x* = 0 into Equations (7) and (8), respectively, the integral constant is:

(9) $$C=\frac{qL^{3}}{6}\;\;D=-\frac{qL^{3}}{24}$$

Then, the angle equation and deformation equation of the driving model are obtained as follows:

(10) $$\theta \left(x\right)=-\frac{q}{6EI}\left[{\left(L-x\right)}^{3}-L^{3}\right]$$

(11) $$w\left(x\right)=\frac{q}{24EI}\left[{\left(L-x\right)}^{4}+4L^{3}x-L^{4}\right]$$

**Figure 3:**
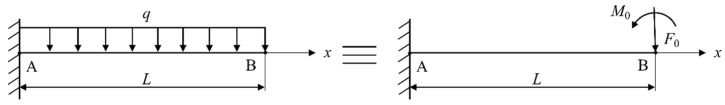
Equivalent mechanical model of the IPMC load mechanism.

The cantilever beam model subjected to a uniform load can be further simplified to a cantilever beam model, subjected to tip load *F*_0_ and bending moment *M*_0_ at the free end, as shown in Figure 3 below.

In this simplification process, the equivalent tip load *F*_0_ and bending moment *M*_0_ are expressed as:

(12) $$F_{0}=qL$$

(13) $$M_{0}=F_{0}\frac{L}{2}$$

The blocking force *F*_B_ is proportional to the input voltage *U* [21], then:

(14) $$F_{B}=\alpha U$$

Combining Equations (12)–(14), the blocking force *F*_B_ can be expressed as:

(15) $$F_{B}=\frac{3qL}{8}\Rightarrow qL=F_{0}=\frac{8}{3}\alpha U$$

where α is the exchange charge density per unit:

(16) $$\alpha =-\frac{EI}{R_{1}E_{1}bh_{1}(h_{1}+h_{2})}$$

where *b* is the width of the IPMC and *h*_1_ and *h*_2_ represent the thickness of the electrode layer and substrate of IPMC, respectively.

Under voltage excitation, the farthest deformation and angle of the free end of the cantilever beam reach the maximum value, and combined with Equation (3), the relationships between the maximum deflection angle and the maximum output deformation and the photovoltaic voltage are expressed as follows:

(17) $$w_{\max}=\frac{\alpha L^{3}}{3EI}(1-e^{-\frac{t}{\tau^{\prime }}}){U_{s}}^{\prime }$$

(18) $$\theta_{\max}=\frac{4\alpha L^{2}}{9EI}(1-e^{-\frac{t}{\tau^{\prime }}}){U_{s}}^{\prime }$$

where *EI* is the bending stiffness of the IPMC.

## 3. Experiments and Analysis

**Figure 4:**
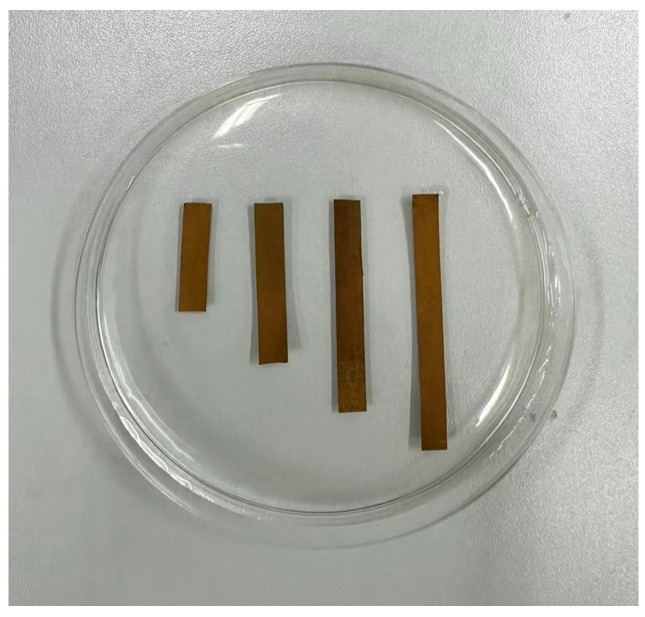
IPMC sample.

In order to verify the feasibility of an IPMC optical-controlled flexible driving method based on PLZT ceramic, a series of driving performances of IPMC under a direct current and light excitation were carried out. With different IPMC sizes, the input voltage and the light intensity as variables, the variation trend of the IPMC output deformation was measured. In this article, the metal electrode layer of the IPMC was Pt, the surface resistance of the material was small, and the property was stable. IPMC lengths of 20 mm, 30 mm, 40 mm and 50 mm were selected, respectively, and were named 20-IPMC, 30-IPMC and so on, according to their length. The width was 5 mm and the thickness was 0.2 mm, as shown in Figure 4.

### 3.1. Analysis of Direct Current Driving

**Figure 5:**
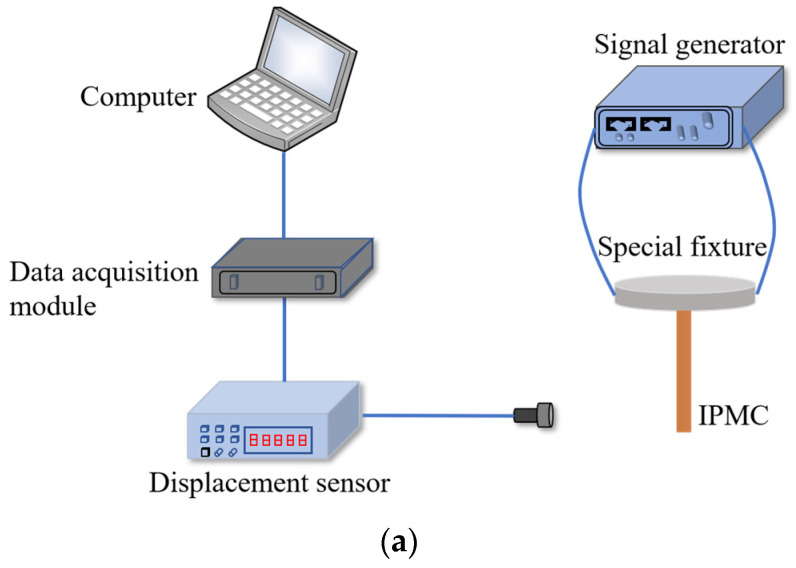

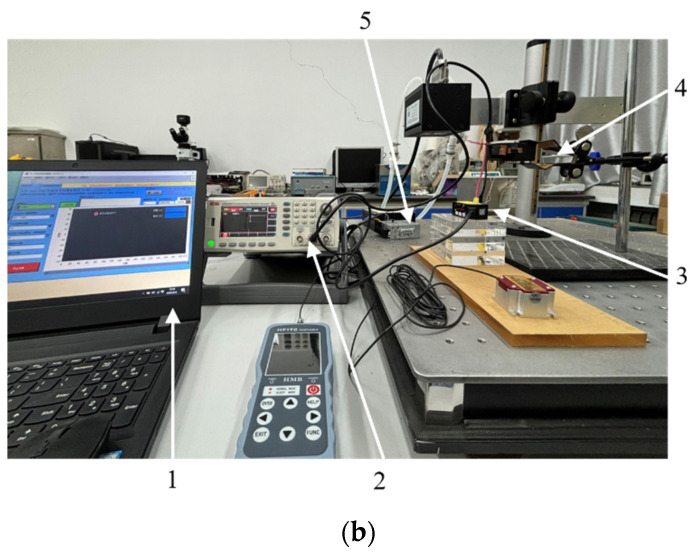
The diagram of direct current driving experimental setup. (**a**) Experimental schematic diagram. (**b**) The experimental test platform. (1) Computer, (2) Signal generator, (3) Displacement sensor, (4) Special fixture, (5) Data acquisition module.

Figure 5 shows a diagram of the direct current driving experimental setup. The signal generator (Agilent33522A Keysight: Los Angeles, CA, USA) has a dual-channel function and its output amplitude ranges from 0 to 10 V. The displacement sensor (HG-C1050, Panasonic: Osaka, Japan) has a measuring range of ±15 mm and an accuracy of 30 μm. The data acquisition module has a four-channel single-ended input function, with a 16-bit effective resolution A/D, and can convert data into digital signals for storage.

One end of the IPMC is fixed onto the special fixture. The signal generator, respectively, outputs 1~4 V direct current (node is 0.5 V), which is the input voltage of the IPMC driving; the output deformation is monitored in real time by the micro laser displacement sensor, collected and uploaded to the PC by the data acquisition module. After each test is completed, the IPMC is placed in pure water for 5~10 min to avoid long-term water loss affecting the next test.

**Figure 6:**
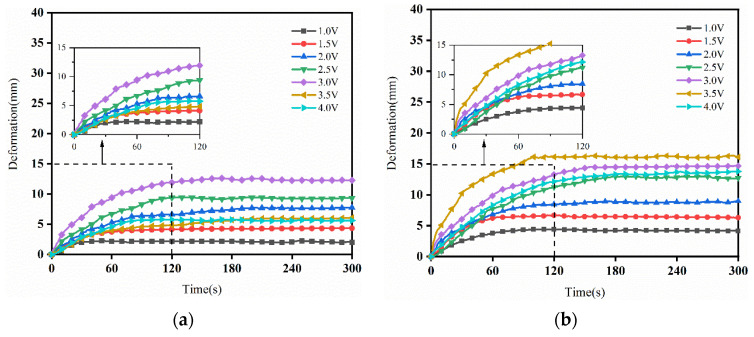

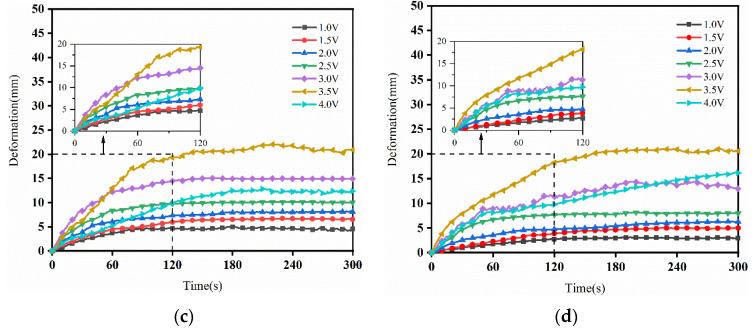
The deformation curves of IPMC under different voltages: (**a**) 20-IPMC, (**b**) 30-IPMC, (**c**) 40-IPMC, (**d**) 50-IPMC.

Figure 6 shows the deformation curves of the IPMC under different voltages. Figure 6a shows the deformation curve of 20-IPMC: when the input voltage was 1–3 V, the deformation increased with the increasing voltage; when the voltage was 3 V, its deformation reached the maximum. Figure 5b–d shows the deformation curves of 30-IPMC, 40-IPMC and 50-IPMC: when the input voltage was 1–3.5 V, the deformation of IPMC increased with increases in the voltage, and the deformation reached the largest value when the voltage was 3.5 V.

**Figure 7:**
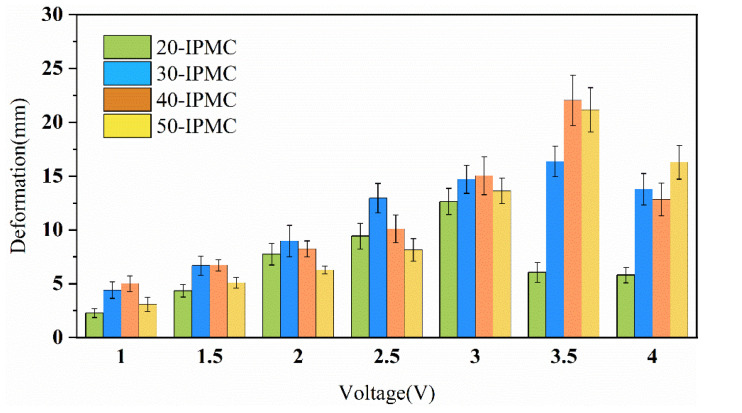
Comparison of the maximum deformation of IPMC under different voltages.

From Figure 6, under the action of different voltage excitations, the output deformations of different IPMC sizes showed a similar trend: within 300 s, the output deformations gradually increased and eventually stabilized over time. From the point of view of the response speed, each output deformation went through a process of rapid growth, before slowing down and finally reaching a stable state; when the driving voltage was 3.5 V, 20-IPMC, 30-IPMC, 40-IPMC and 50-IPMC all reached the maximum deformation within 100–160 s. Combined with the above analysis of the output deformation and response time of the IPMC at different sizes, the 40-IPMC showed the largest output deformation, a faster response speed and a longer stable operating time. Figure 7 shows a comparison of the maximum deformation of IPMC under different voltages.

From Figure 7, the similar voltage dependence is presented, regardless of the size of the IPMC. As the voltage increased, the deformation of IPMC initially increased, and then stabilized. In particular, when the voltage was 3.5 V, the 40-IPMC exhibited the largest deformation. Thus, under a specific voltage, the response of the IPMC with different sizes to the voltage follows certain rules and has a certain stability.

**Figure 8:**
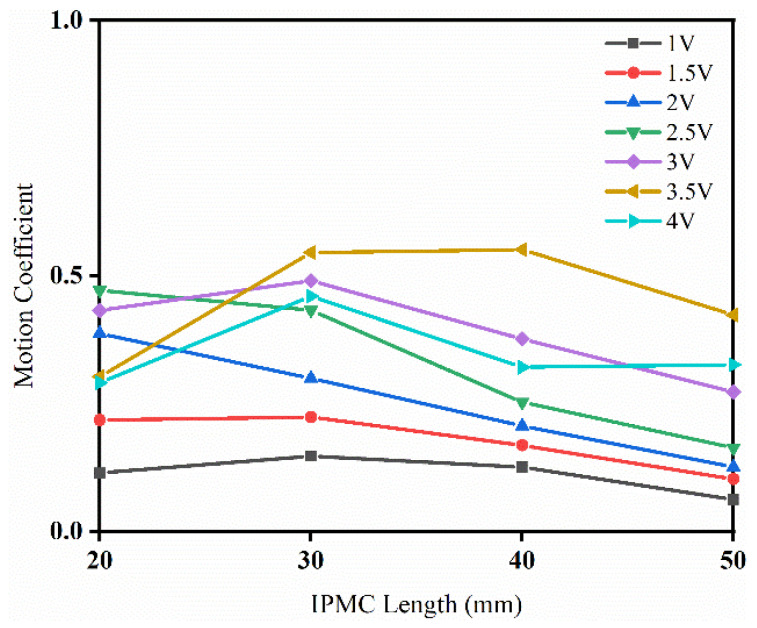
The motion coefficient of the IPMC under different voltages.

However, for IPMC flexible driving, its deformation is closely related to the length of the material; that is, an IPMC with a large length has a large curvature radius, and its output deformation must be large. Therefore, simply discussing the maximum deformation cannot accurately reflect the driving ability of the IPMC. To evaluate the driving performance of the IPMC more accurately, the ratio of the maximum deformation to the material length is defined as the motion coefficient. Under the excitation of different voltages, the calculation results of IPMC motion coefficients of different lengths are drawn as in Figure 8.

It is obvious from the analysis in Figure 8, that compared to 20-IPMC and 50-IPMC, 30-IPMC and 40-IPMC show better overall driving performance. The driving performance of IPMC did not increase linearly with size; that is, it is not the case that a longer IPMC material means better motion performance, but that there is an optimal ratio between the length and deformation.

### 3.2. Analysis of Optical-Controlled Driving

Under the irradiation of an ultraviolet light source, PLZT ceramic can transfer light energy into electrical energy due to its photovoltaic characteristics—thus generating a photovoltaic voltage, which is the driving voltage of the IPMC load mechanism. Under the action of this voltage, the IPMC load mechanism produces a large angle deformation based on the actuating effect, and its output characteristics can be further studied.

**Figure 9:**
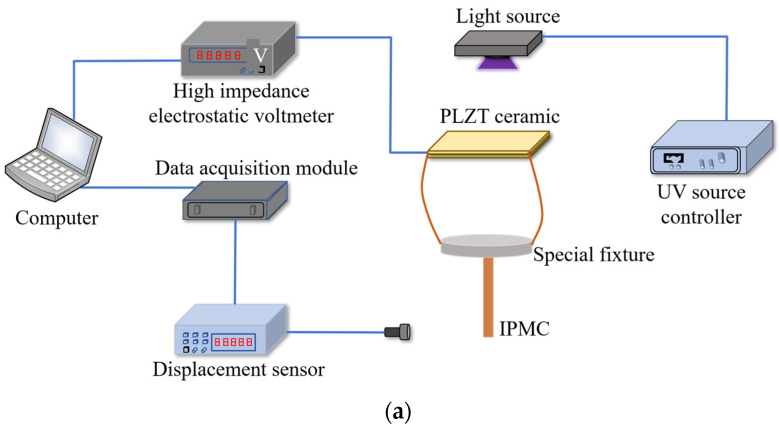

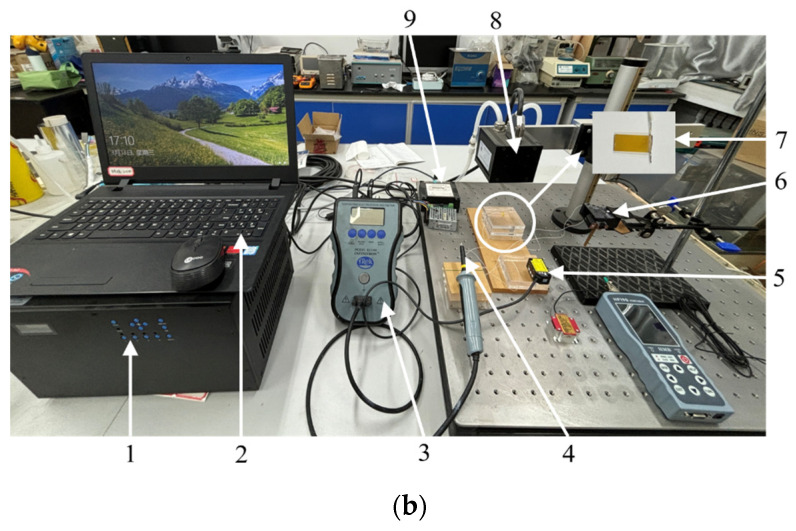
Diagram of the optical-controlled driving experimental setup. (**a**) Experimental schematic diagram. (**b**) The experimental test platform. (1) UV source controller, (2) Computer, (3) High-impedance electrostatic voltmeter, (4) Sensor head of high-impedance voltmeter, (5) Displacement sensor, (6) Special fixture, (7) PLZT ceramic, (8) Light source, (9) Data acquisition module.

Figure 9 shows a diagram of the optical-controlled driving experimental setup. The irradiation area of the light source (LED-UV) is 30 mm × 20 mm and the light source wavelength is 365 nm; the light intensity and irradiation time are controlled by a UV source controller. The high-impedance electrostatic voltmeter (Trek Model: 821 HH) has a measuring range of 0 ± 2 kV, and the measuring accuracy is ±1% of the maximum display output.

The UV light source controller controls the high-energy UV light source to irradiate vertically and uniformly on the PLZT ceramic surface, which is connected to the high-impedance voltmeter sensor probe via a silver wire; the voltage data received by the sensor probe is transmitted to the high-impedance voltmeter controller and finally to the PC. Meanwhile, the output deformation signal of the IPMC is collected and transmitted to the computer. The size parameters of the PLZT ceramic are 10 mm × 5 mm × 0.8 mm (length × width × thickness). Similarly, after each test is completed, the IPMC is placed in pure water for 5–10 min to avoid long-term water loss.

**Figure 10:**
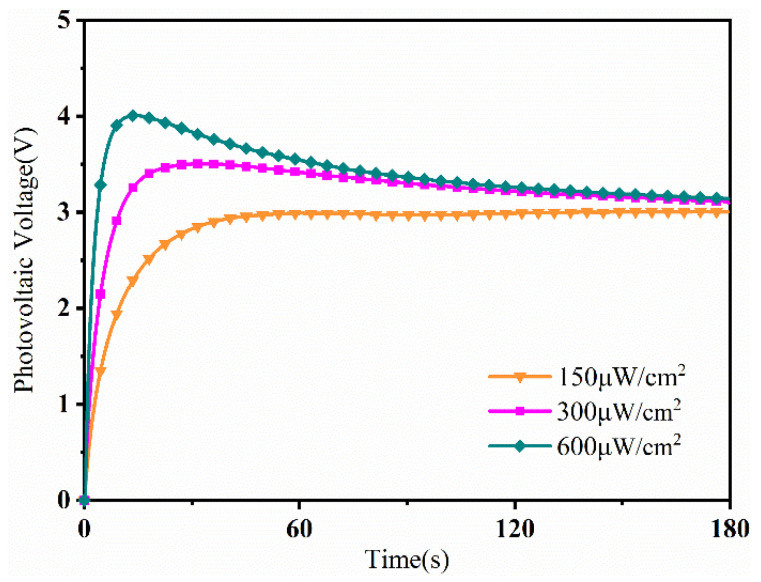
The photovoltage voltage under irradiation with different light intensities.

Light intensities of 150 μW/cm^2^, 300 μW/cm^2^ and 600 μW/cm^2^ were applied, Figure 10 shows the photovoltaic voltage curves of the PLZT ceramic under different selected light intensities. Under the light excitation, the photovoltaic voltage generated by the PLZT ceramic quickly rose to a saturated state, and then maintained a stable state. The saturation value of the photovoltaic voltage gradually increased with increases in the light intensity, the response speed also increased, and the time required to reach the saturation value decreased.

**Figure 11:**
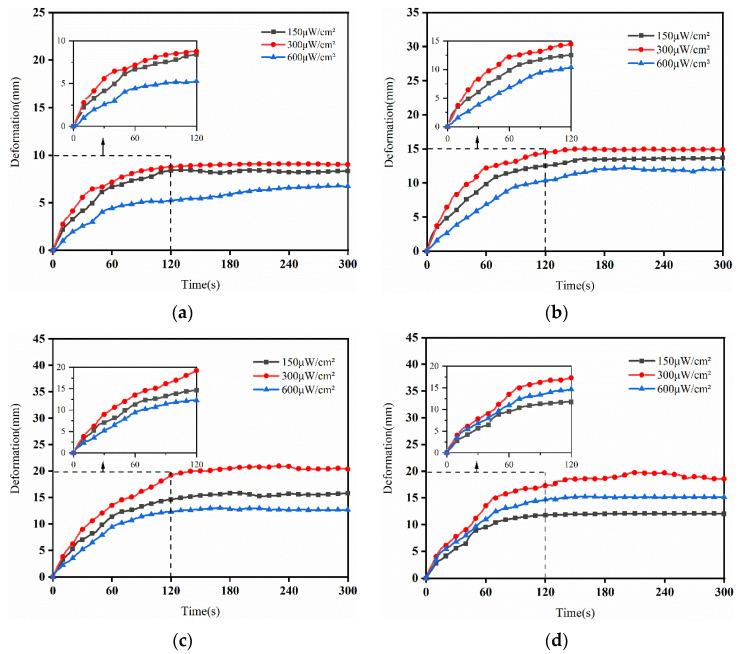
The deformation curves of the IPMC under different light intensities: (**a**) 20-IPMC, (**b**) 30-IPMC, (**c**) 40-IPMC, (**d**) 50-IPMC.

**Figure 12:**
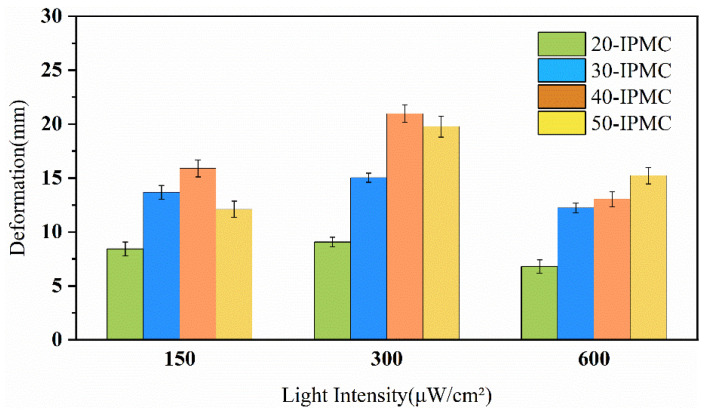
Comparison of the maximum deformation of the IPMC under different light intensities.

Figure 11 shows the deformation curves of the IPMC under different light intensities. According to the analysis in Figure 11, the output deformations of the different IPMC sizes under the excitation of a light source show a similar trend: the deformations gradually increased and reached a stable state. When the light intensity was 300 μW/cm^2^, all sizes of IPMC showed the maximum output deformation, and reached this value in 90–120 s. Furthermore, integrated analysis of the deformation and response time, showed that when the light intensity was 300 μW/cm^2^, the 40-IPMC had the largest deformation, a faster response speed and a longer stable operating time. Meanwhile, from the theoretical analysis, it can be seen that the deformation under different light intensities conformed to the trend predicted by the mathematical model, and tended to be stable after reaching the saturation value. Figure 12 shows a comparison of the maximum deformation of the IPMC under different light intensities.

**Figure 13:**
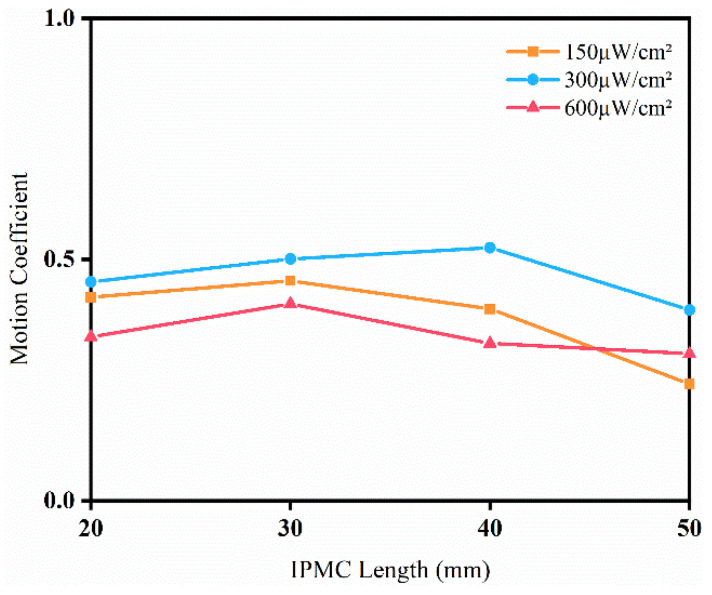
The motion coefficient of the IPMC under different light intensities.

From Figure 12, it is obvious that all the IPMCs showed a similar dependence on the applied light intensity amplitude; that is, the driving voltage increased when the input light intensity increased, and the deformation also increased, and finally reached a stable state. In addition, when the light intensity was 300 μW/cm^2^, the 40-IPMC showed the greatest deformation compared to other sizes of IPMC. Similarly, the motion coefficient of IPMC under different light intensities are drawn as Figure 13.

From Figure 13, it can be noticed that the results were similar to those of the direct current driving: 30-IPMC and 40-IPMC exhibited better driving performance, and the performance of the IPMC driven by light energy did not linearly improve with increases in size.

Synthesizing the above analysis, from the experimental results of the driving performance of IPMC under light intensities, the different sizes of IPMC showed a similar deformation trend under different light intensities. Compared with the performance of the IPMC driven by a direct current, the IPMC driven by light energy also had good driving characteristics, which also verifies the effectiveness and rationality of the IPMC optical-controlled flexible driving method based on PLZT ceramic.

## 4. Conclusions

In this paper, an IPMC optical-controlled flexible driving method based on PLZT ceramic was proposed; the mathematical model was conducted on the basis of the photovoltaic characteristics of PLZT ceramic, and the analysis of its driving performance and motion coefficient was investigated by a series of experiments. The results show that under illumination by a light source, compared with the direct current driving method, IPMC also has excellent driving performance and a fast response speed. Furthermore, the performance of IPMC stimulated by light energy also did not increase with increases in size; that is, there is a motion coefficient. According to the above analysis, the proposed optical-controlled flexible driving method can not only make up for the limitation of non-independence in the original IPMC driving method, but also lay a foundation for the practical engineering application of the IPMC optical-controlled flexible driving method based on PLZT ceramic.

## Data Availability

Data are contained within the article.
